# A cross-sectional survey of *Aedes aegypti* immature abundance in urban and rural household containers in central Colombia

**DOI:** 10.1186/s13071-017-2295-1

**Published:** 2017-07-27

**Authors:** Hans J. Overgaard, Víctor Alberto Olano, Juan Felipe Jaramillo, María Inés Matiz, Diana Sarmiento, Thor Axel Stenström, Neal Alexander

**Affiliations:** 1 Faculty of Science and Technology, Norwegian University of Life Sciences, Ås, Norway; 2 Instituto de Salud y Ambiente, Universidad El Bosque, Bogotá, Colombia; 3 SARChI, Institute for Water and Waste Water Technology, Durban University of Technology, Durban, South Africa; 4 MRC Tropical Epidemiology Group, London School of Hygiene and Tropical Medicine, London, UK

**Keywords:** *Aedes aegypti*, Immature stages, Household water container, Dengue, Mosquito

## Abstract

**Background:**

*Aedes aegypti*, the major vector of dengue, breeds in domestic water containers. The development of immature mosquitoes in such containers is influenced by various environmental, ecological and socioeconomic factors. Urban and rural disparities in water storage practices and water source supply may affect mosquito immature abundance and, potentially, dengue risk. We evaluated the effect of water and container characteristics on *A. aegypti* immature abundance in urban and rural areas. Data were collected in the wet season of 2011 in central Colombia from 36 urban and 35 rural containers, which were either mosquito-positive or negative. Immature mosquitoes were identified to species. Data on water and container characteristics were collected from all containers.

**Results:**

A total of 1452 *Aedes* pupae and larvae were collected of which 81% were *A. aegypti* and 19% *A. fluviatilis*. *Aedes aegypti* immatures were found in both urban and rural sites. However, the mean number of *A. aegypti* pupae was five times higher in containers in the urban sites compared to those in the rural sites. One of the important factors associated with *A. aegypti* infestation was frequency of container washing. Monthly-washed or never-washed containers were both about four times more likely to be infested than those washed every week. There were no significant differences between urban and rural sites in frequency of washing containers. *Aedes aegypti* immature infestation was positively associated with total dissolved solids, but negatively associated with dissolved oxygen. Water temperature, total dissolved solids, ammonia, nitrate, and organic matter were significantly higher in urban than in rural containers, which might explain urban-rural differences in breeding of *A. aegypti*. However, many of these factors vary substantially between studies and in their degree of association with vector breeding, therefore they may not be reliable indices for vector control interventions.

**Conclusions:**

Although containers in urban areas were more likely to be infested with *A. aegypti*, rural containers still provide suitable habitats for *A. aegypti*. Containers that are washed more frequent are less likely to produce *A. aegypti.* These results highlight the importance of container washing as an effective vector control tool in both urban and rural areas. In addition, alternative designs of the highly productive washbasins should continue to be explored. To control diseases such as dengue, Zika and chikungunya, effective vector breeding site control must be implemented in addition to other interventions.

## Background

Arboviruses, such as dengue, Zika and chikungunya, are transmitted by mosquitoes of the genus *Aedes*, especially *Aedes aegypti* (L.). This species preferentially breeds in man-made water containers in close proximity to human habitations [1]. The risk of dengue transmission increases with rapid, unplanned, and unregulated urban development, poor water storage practices, and unsatisfactory sanitary conditions [1]. These factors are likely to affect the risk of Zika and chikungunya transmission, although the former can also be transmitted sexually [2]. Dengue fever is rapidly spreading globally [3]; approximately 2.5 billion people live in risk areas and an estimated 390 million infections occur annually in more than 100 countries [3]. The annual number of deaths from dengue has been estimated at ~22,000, mainly among children [4]. Zika has received much attention due to recent outbreaks, starting in Brazil in 2015, resulting in microcephaly in babies. Zika is continuously spreading to areas with competent vectors and currently, at least 84 countries and territories have reported vector-borne Zika virus transmission [5]. The first Zika cases recorded in Colombia were in 2015, when more than 11,700 cases were notified (Boletín Epidemiológico Semanal, Instituto Nacional de Salud, http://www.ins.gov.co). Chikungunya has been identified in over 60 countries, with recent outbreaks in the Indian Ocean, India, southeast Asia and Latin America [6]. Chikungunya can cause severe joint pains, with fever, muscle pain, headaches and other symptoms. In Colombia, local transmission of chikungunya was first identified in 2014, followed by an outbreak with close to 100,000 people being infected and at least eight deaths recorded [7].

Dengue is generally considered an urban disease, but is also of importance in rural areas (e.g. [8, 9]). In Latin-America, *A. aegypti* is expanding into peri-urban and rural areas [10–16]. *Aedes aegypti* has a very limited flight range [17], therefore mosquitoes most likely disperse passively along human transportation networks, e.g. hitchhiking in cars, buses and boats [14]. A distinction between disease in urban and rural areas is often not apparent. A person might become infected in a rural area, but disease symptoms can appear and diagnosis be made in an urban area and *vice versa*. Regular human movement between rural and urban areas compounds disease records and source of infection. Definitions of “urban” and “rural” differ in both time and place and may not be directly comparable, for example as illustrated by the Urban-Rural Classification Scheme for Counties US National Center for Health Statistics [18] or the WHO/UNICEF Joint Monitoring Programme (JMP) which monitors annual progress on sanitation and drinking water [19].

Mosquito productivity depends on various factors, such as the nutritional quality of the larval environment [20], container type [21, 22], surrounding environmental conditions [23], and climate and seasonality [24, 25]. In addition, socioeconomic factors, such as household size, income, education, water storage practices, and solid waste management, may affect vector presence and abundance [26, 27]. Mosquito abundance is potentially lower in higher socioeconomic strata than in lower strata [26] and in premises in good conditions (assessed by house condition, tidiness of the yard, and degree of shading) [28, 29].

A higher number of dengue cases in urban compared to rural areas may be explained by differences in human and mosquito population densities and higher likelihood of human-mosquito contact (e.g. discussed in [8]). However, higher risk in urban settings could also be partly explained by differences in container characteristics and domestic water management and hence mosquito productivity. In 2012, an estimated 79% of the world’s urban population had piped water into their houses, compared to only 33% in rural areas [30], suggesting that a higher proportion of rural households store water than urban households. It is not clear whether there are differences in mosquito productivity between urban and rural containers. Knowing this could help authorities plan control activities.

In Colombia, *A. aegypti* is present in all departments (first-level administrative subdivision) and up to an altitude of at least 2300 m above sea level [31]. The most productive and permanent containers are ground tanks and concrete washbasins for laundry (*albercas*), whereas other containers, such as bottles, cans, tires, etc., are only productive during the rainy season and produce low numbers of pupae [21, 32, 33]. In Colombia, larval control, such as the application of the organophosphate temephos, is generally only practiced in epidemic situations. For routine control, communities are recommended to keep containers covered and clean [34].

Mosquito oviposition behavior is affected by visual, tactile, and olfactory cues, including physico-chemical properties of the water [35]. Oviposition by female *A. aegypti* is often argued to take place in containers with clean water; however, this is not always true as *A. aegypti* has been found to breed in polluted water and raw sewage [36, 37]. The number of immatures per container is not homogeneously distributed [38], indicating that female mosquitoes prefer to oviposit in specific types of containers. Such containers may be more epidemiologically important than other container types [38, 39]. The typical skip oviposition behavior observed in female *A. aegypti* [40] may be modulated by the presence and abundance of conspecifics [41, 42] and by the availability of breeding sites [43]. Larval mosquito diets mainly consist of bacteria and detritus [44, 45]. Availability of nutrition in containers is critical for mosquito development and may affect mosquito size and survival [46, 47], which in turn may affect vector-borne disease transmission outcome [48, 49]. For example, *A. aegypti*-positive containers have been found to have more dissolved nitrogen ions than negative containers [50]. It is likely a combination of the oviposition behavior of female mosquitoes and the quality of the breeding habitat that influences the importance of certain containers over others.

The objectives of this study were to determine whether *A. aegypti* immature production differed in urban and rural areas and what factors were associated with *A. aegypti* immature production in mosquito-infested containers in urban and rural areas.

## Methods

### Study area

**Fig. 1:**
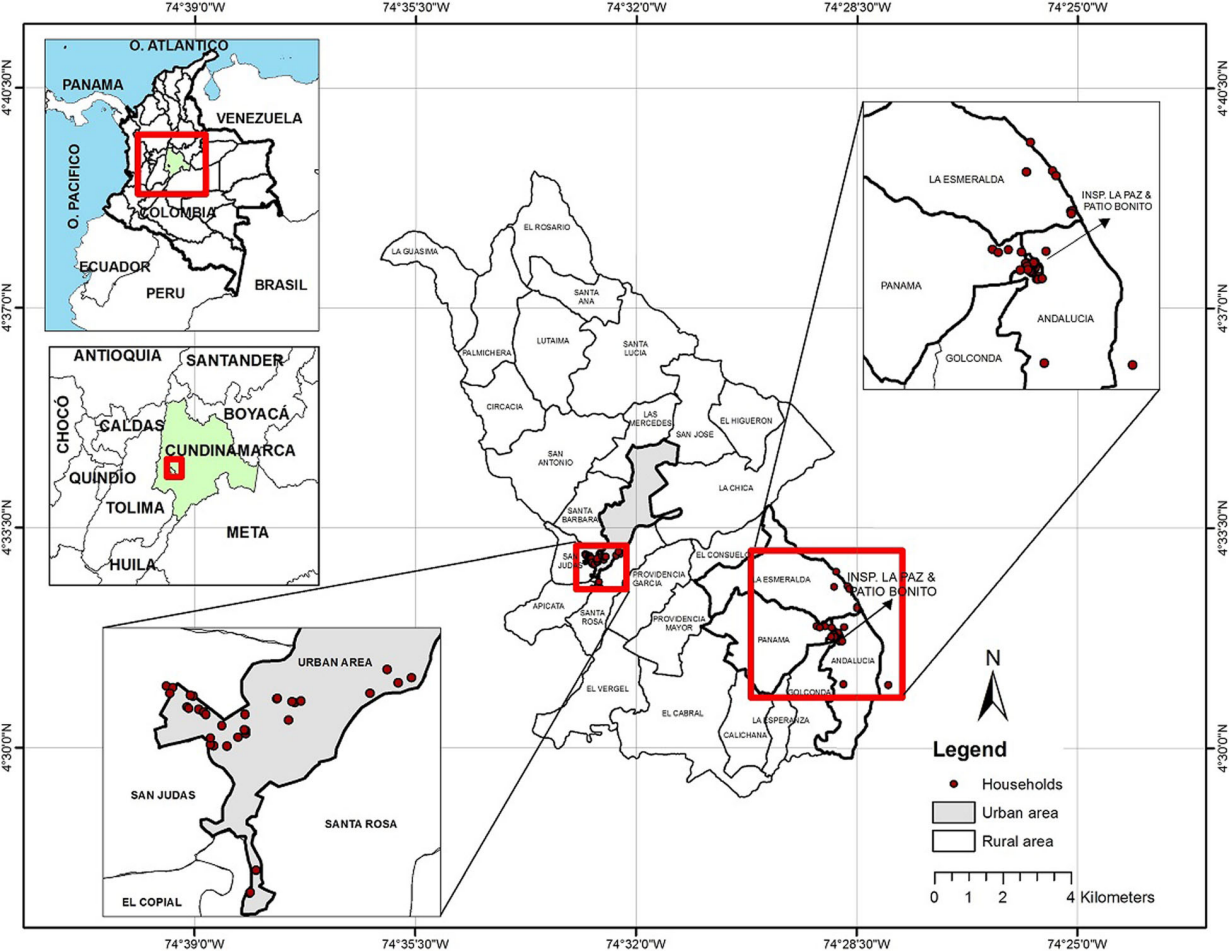
Location of collection households (*red spots*) in urban (lower left inset) and rural (upper right inset) areas in Anapoima municipality (main central map), Cundinamarca Department, Colombia

The study was carried out in urban and rural sites in Anapoima municipality, Cundinamarca, central Colombia (centered at 4.551271N, 74.536436W) (Fig. 1). In 2011, Anapoima had a projected population of approximately 12,500 inhabitants (based on the general census in 2005), with 57% in rural areas [51]. The total population in Anapoima town (*cabecera municipal*) in 2011 was ~5300 with a population density of ~739 persons/km^2^. The corresponding figures for the rural area were ~7100 and ~54 persons/km^2^. The total municipal area is 124.2 km^2^ at an average altitude of 700 m above sea level (masl), an average annual temperature of 26 °C and rainfall of 1300 mm. The main economic activities are agriculture (sugar cane, coffee, fruit and livestock) and tourism. The natural vegetation consists of dry tropical forest, premontane and lower montane moist forests.

**Fig. 2:**
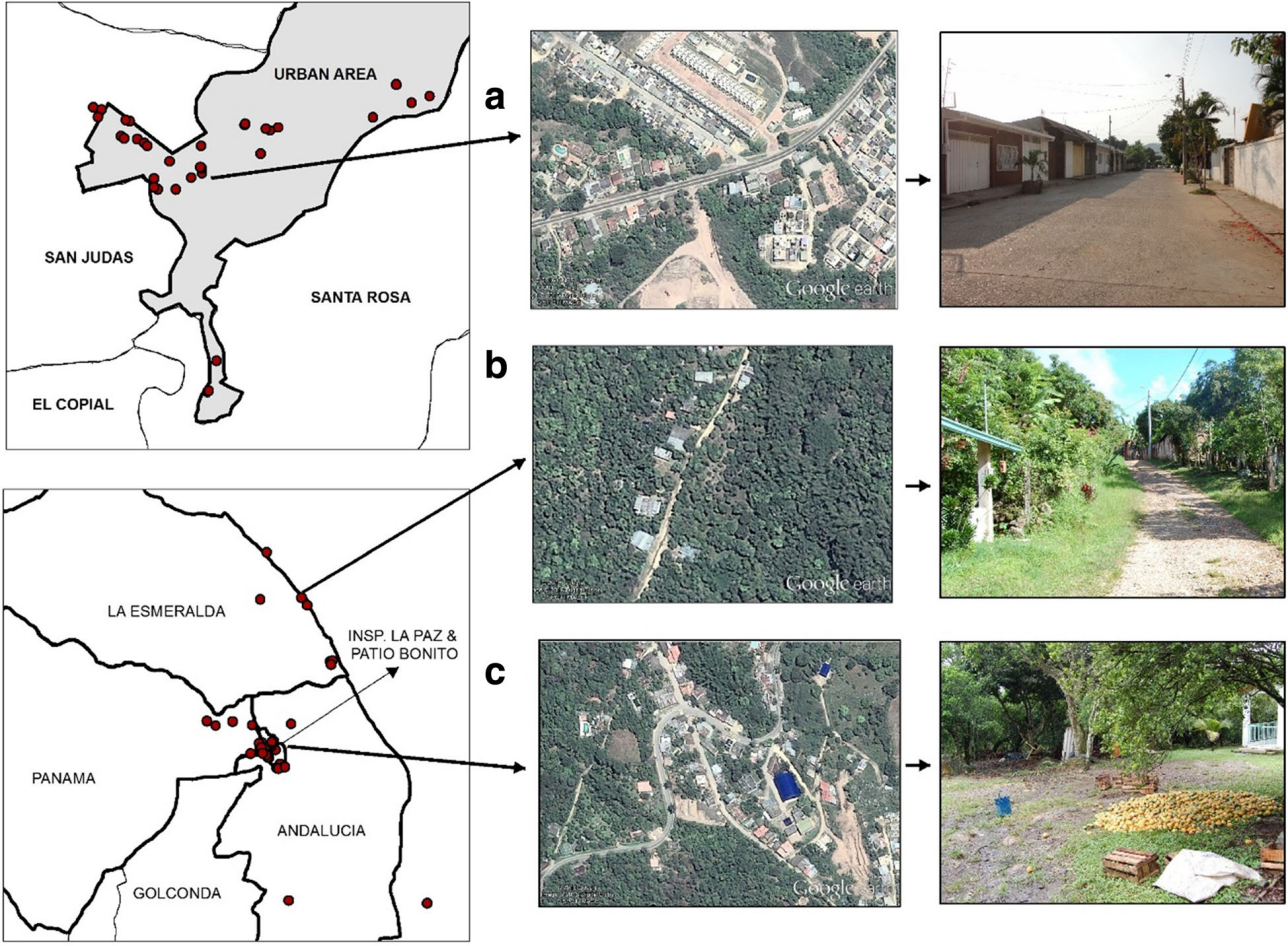
Examples of environments surrounding households in the urban area (**a**) and the rural area (**b** and **c**), Anapoima municipality, Cundinamarca Department, Colombia

Official criteria from the National Administrative Department of Statistics, Colombia were used to distinguish between urban and rural study sites [52]. An urban area, according to these criteria, is characterized by buildings and adjacent structures grouped into blocks bounded by streets and avenues; the presence of essential public services such as water, sewage, electricity, hospitals, schools; and the seat of the municipal administration. A rural area is characterized by housing and dispersed farms without a layout of streets, generally lacking of public services and other facilities featured in urban areas. Rural areas also include so-called *inspecciones*, which are small concentrations of houses more densely populated than the dispersed areas. The rural area in Anapoima has a lower socio-economic level than the urban area, with 36% of the population classified as poor and 13% of those >15 years old being illiterate; the corresponding figures for the urban area were 21 and 5%, respectively [53]. The criteria used to define poverty were if people lived in overcrowded conditions (> 3 people/room), without piped water connection or sanitary facilities, in households with high economic dependence (i.e. > 3 persons/employed household member), low education level of household head (< 2 years of primary education), and children aged between 6 and 12 years not attending school [53]. Aerial images from Google Earth and environments surrounding some of the study households are shown in Fig. 2.

Study sites within the urban and rural areas were selected based on *A. aegypti* infestation registers from Anapoima Secretary of Health and unpublished data of the Instituto de Salud y Ambiente, Universidad El Bosque (Informe Final. Resultados del diagnóstico del proyecto Prevención del dengue y control del *A. aegypti* en el área rural del municipio de Anapoima, Cundinamarca, 2011). Sites with highest infestation rates in the urban area were selected; in 2010 those sites had a Container Index of 14.3% and a Breteau index of 15 (pers. comm. José Fernando Sánchez. Coordinador Programa ETV y Zoonosis, Secretaría de Salud, Gobernación de Cundinamarca). Thus, six neighborhoods were selected in the urban area (Centro, San José, Las Palmas, Asopobin, La Estrella and Nueva Colombia). These six neighborhoods had a total of 292 households at the time of the study. In the rural study area, three adjacent sites were selected: Inspección La Paz (total 52 households or 26 houses), Patio Bonito (total 45 households or 23 houses), and Andalucía (total 143 houses). Entomological data from 2010 collected in the rural areas of Anapoima showed a House Index of 5% in Andalucia and 18% in La Paz and Patio Bonito each. The average altitude was 697 (range: 636–743) masl for the urban households and 1038 (range: 941–1124) masl for the rural ones. Routine larval control consists of cleaning and covering of containers, whereas temephos is applied during epidemics [34].

### Study design and sample size

This is a cross-sectional study with the water storage container as the unit of analysis. The sample size was determined to be at least 34 containers representing the urban and rural area, respectively (a total of at least 68 containers) giving 80% power to detect a ratio of means of 2, with negative binomial *k* parameter of 2 and significance level of 5% [54]. In the urban site, 33 of 292 houses were selected for collections. In the rural sites, 37 of 192 houses were selected, 29 in Patio Bonito and 8 dispersed houses in La Paz and Andalucía combined.

### Data collection

Field staff randomly selected houses in the two areas to complete the calculated container sample size. The aim was to sample one mosquito-positive and one mosquito-negative container from each selected household. All containers were inspected visually, and each was considered positive if either larvae or pupae were present. Then, in households with at least one mosquito-positive and at least one mosquito-negative container, one of each type was sampled. In households with only positive containers, one container was sampled. If two or more containers were positive, then the one with highest mosquito infestation was selected, based on visual inspection of the presence of larvae and pupae. In households with only negative containers, the first available container was sampled. Eggs were not included in the collection framework. Data on the total number of containers present in each inspected household and the number of positive containers in each household were not collected. Data were only collected from two types of containers, ground tanks (low tanks) and concrete washbasins for laundry (*albercas*), because these are the most important and productive containers identified in Colombia [33]. Based on our unpublished data from rural areas in Anapoima, we also found the same types of containers being the most infested (Instituto de Salud y Ambiente, Universidad El Bosque. Informe Final. Resultados del diagnóstico del proyecto Prevención del dengue y control del *A. aegypti* en el área rural del municipio de Anapoima, Cundinamarca, 2011).

Before inspecting containers, residents were first asked if any container had been treated in any way. None of the collection containers were reported as being treated by any chemical or cleaned during the last 72 h. In total, 71 domestic water containers were included, of which 37 were mosquito-positive and 34 mosquito-negative. Of the 71 containers, 36 were located in the urban area and 35 in the rural area. Collections were carried out in October–December 2011 (rainy season). In addition, the altitude of the household was measured using a GPS handheld unit.

#### Mosquitoes

All mosquito immatures (pupae and larvae) of the genus *Aedes* were collected from the identified positive containers using glazed soup ladles or sweep nets. In addition, a sample of immatures identified to other genera were collected using ten dips from different parts of the container, the total number of larvae counted, and approximately 10% stored for later identification. Collected larvae and pupae were kept in plastic vials with 70% ethanol. Taxonomic identification of mosquitoes was carried out in the entomology laboratory of the Lazos de Calandaima Foundation in Anapoima using appropriate taxonomic keys [55–57].

#### Container characteristics

Data on the following container characteristics were collected: site (rural, urban); type (ground tank, wash basin); location (outdoors, indoors); material (plastic, cement, metal); immediate water source (village pipe, municipal pipe, rain only, rain + village pipe, rain + municipal pipe, village pipe + river + spring); use (washing + cleaning, multiple uses including drinking, multiple uses excluding drinking, drinking only, plants); lid on container (none, effective lid, ineffective lid); reported frequency of washing (weekly, monthly, never); whether in shade (yes, partial, no); and whether under roof (yes, partial, no).

#### Water characteristics

For each container, data on water temperature (°C), pH, electrical conductivity (EC, μS/cm) and total dissolved solids (TDS, mg/l) were collected in situ using a HACH multiparameter (sensION+ Portable pH & EC field kit, with MM150 meter, 5059 electrode). Thereafter, a composite water sample consisting of 2–6 subsamples (depending on water level), including central and peripheral parts of containers and biofilms on container walls, amounting to 300–2000 ml, was taken from each container. Samples were stored on ice and conserved according to prescribed methods [58]. The content of ammonia (NH_4_^+^, mg/l), phosphate (PO_4_^3-^, mg/l), nitrate (NO_3_^-^ mg/l), dissolved oxygen (DO, mg/l), and total suspended solids (TSS, mg/l) were analyzed by Daphnia Laboratory, Bogotá, Colombia (certified laboratory by IDEAM, Ministry of Environment and Sustainable Development, Res. 0347/2010 and 0710/2012). The visible presence of algae (yes/no), organic matter (leaves, etc.) (yes/no), and macroinvertebrates (unspecified aquatic insects, crustaceans, etc.) (yes/no) was assessed in the field.

Water samples for bacterial abundance assessment were prepared in the laboratory for subsequent microbial counts using fluorescent microscopy. Briefly, three sample dilutions (1:10, 1:20, 1:40) were prepared to a final volume of 100 ml. Two duplicates of 10 ml of each dilution were placed in 27-well glass plates, passed over a flame for fixing bacteria and stained with 1000 mg/ml acridine orange solution for 3 min. An oil immersion of samples were observed under 100× objective under UV light with a fluorescence microscope (A2 AxioVision Carl Zeiss Microscopy, LLC). Photos of 20 microscope fields per well for each replica were taken and the number of bacterial cells per photo were determined by indirect counting using Scion Image program (Beta 4.0.2, Scion Corporation) accounting for well area, dilution and initial volume. Results were expressed as the total number of bacterial cells per milliliter of sample. Fluorescent microscopy was done in the Laboratory of Virology, Universidad El Bosque, Bogotá, Colombia.

### Data analysis

Descriptive analyses were used to explore the data. Analysis of abundance was based only on mosquito-positive containers. Mosquito-negative containers are not informative on abundance since the study design ensured that their number equaled that of the mosquito-positive containers. A zero-truncated negative binomial model was used to compare *A. aegypti* larval abundance between urban and rural sites. This model was chosen because all mosquito-negative containers had zero larvae. Differences in *A. aegypti* pupal abundance in mosquito-positive containers between sites were analyzed using a standard (non-truncated) negative binomial model, because some positive containers did not have pupae. A zero-truncated negative binomial model was also used to compare larval abundance of non-*Aedes* species, mainly *Culex* species, between urban and rural sites.

Before logistic regressions were carried out, non-normally distributed continuous variables were log_10_-transformed, then bivariate analyses, using Chi-square test and Student’s t-test for categorical and continuous variables, respectively, were used to select variables (with *P* < 0.1). Pearson’s correlation coefficients were then calculated among remaining variables to assess collinearity. For variables with a correlation > 0.5 only one was kept, e.g. logEC was removed due to high correlation with logTDS (*r* = 0.936). Finally, simple and multivariable (backward stepwise) logistic regression analyses were done using container and water characteristics variables as predictors and *Aedes* larvae- or pupae-positivity as response variables. Differences in container and water characteristics between urban and rural sites were analyzed by Chi-square tests and t-tests. Data were analyzed using Stata version 14.1.

## Results

### *Aedes aegypti* productivity in mosquito-positive containers

A total of 1452 *Aedes* immatures (pupae and larvae) were collected of which 1172 (80.7%) were *A. aegypti* and 280 (19.3%) *A. fluviatilis* (Lutz). Other species present in the collected samples were *Culex quinquefasciatus* (Say)*, Cx. coronator* (Dyar & Knab)*, Cx. corniger* (Theobald)*, Limatus durhamii* (Theobald), and other *Culex* spp. not identified to species.

Among positive containers, infestation of *A. aegypti* larvae was 14.3 larvae/container in urban areas and 11.3 larvae/container in rural areas, and this difference was not statistically significant (Table 1). The mean density of *A. aegypti* pupae was higher in urban than in rural areas (6.2 *vs* 1.2 pupae/container), corresponding to a ratio of means more than five times higher (*P* = 0.030, Table 1). In contrast, the mean number of other larvae, i.e. non-*Aedes* larvae (generally *Culex*), was 76% lower than in the rural area (rural: 3.1 larvae/container *vs* urban: 0.6 larvae/container) (*P* = 0.032, Table 1).

**Table 1:** Number of specimens collected in urban and rural containers and ratios of means (95% confidence intervals, CI) from negative binomial regression analyses of mosquito immature abundance in urban compared to rural sites in Anapoima municipality, Colombia

Variable	Level	No. specimens (%)	No. containers	Ratio of means	95% CI	*Z*	*P*
*A. aegypti* larvae	Rural	395 (43)	37	1			
	Urban	514 (57)		1.26	0.48–3.36	0.47	0.639
	Sum	909 (100)					
*A. aegypti* pupae	Rural	41 (16)	18	1			
	Urban	222 (84)		5.13	1.18–22.37	2.18	0.030
	Sum	263 (100)					
Other larvae	Rural	109 (83)	18	1			
	Urban	23 (17)		0.24	0.06–0.89	-2.14	0.032
	Sum	132 (100)					

### Factors associated with *A. aegypti* immature production

Comparing positive and negative containers, the most important factors individually associated with *A. aegypti* presence were frequency of washing the container (*χ* ^2^ = 6.16, *df* = 2, *P* = 0.046), log_10_TDS (*t*_(69)_ = -2.67, *P* = 0.005), DO (*t*_(69)_ = 2.08, *P* = 0.021), and pH (*t*_(69)_ = -1.81, *P* = 0.037). Results from the univariate and multivariate logistic regression models are shown in Tables 2 and 3, respectively. Containers reported to be washed every month or never washed had each four times higher odds of *A. aegypti* infestation compared to containers with a weekly washing (Table 3). In the univariate analysis, TDS was positively associated with *A. aegypti* infestation (univariate: *P* = 0.013), while dissolved oxygen content was negatively associated (univariate: *P* = 0.052; multivariate: *P* = 0.026). pH was not significantly associated with mosquito infestation (univariate: *P* = 0.089). The stepwise multivariate analysis yielded a model with frequency of washing and dissolved oxygen both statistically significantly associated with infestation.

**Table 2:** Odds ratios of factors associated with *A. aegypti* immature infestation in Anapoima municipality, Colombia, using univariate logistic regression (*n* = 71 observations)

Variable	Level	OR (95% CI)	*P*
Frequency of washing container	Weekly	1	
	Monthly	3.45 (1.16–10.29)	0.026
	Never	3.60 (0.93–13.95)	0.064
Total dissolved solids, TDS (log_10_ mg/l)		8.60 (1.57–46.84)	0.013
Dissolved oxygen, DO (mg/l)		0.59 (0.34–1.00)	0.052
pH		2.01 (0.91–4.42)	0.089

*Abbreviations*: *OR* odds ratio; *95% CI* 95% confidence interval

**Table 3:** Odds ratios of factors associated with *A. aegypti* immature infestation in Anapoima municipality, Colombia, using multivariate logistic regression (*R* ^2^ = 0.124; *n* = 71 observations)

Variable	Level	OR (95% CI)	*P*
Frequency of washing container	Weekly	1.00	
	Monthly	4.23 (1.31–13.68	0.016
	Never	4.55 (1.09–18.96)	0.037
Dissolved oxygen, DO (mg/l)		0.51 (0.28–0.92)	0.026

*Abbreviations*: *OR* odds ratio; *95% CI* 95% confidence interval

### Container and water characteristics in urban and rural sites

Container characteristics were similar between the urban and rural sites. Most were washbasins without lids, located outdoors and washed on a weekly or monthly basis. The main significant differences between urban and rural sites were in container materials, source of water, and presence of organic material (see Table 4 for details). The altitude of the rural sites was significantly higher than the urban sites (Table 5). Compared to the rural containers, the water in the urban containers was significantly warmer, had a higher electrical conductivity (EC) and higher concentrations of total dissolved solids (TDS), ammonium and nitrate, but a lower concentration of total suspended solids (TSS) (Table 5).

**Table 4:** Container and water characteristics (categorical variables) in urban and rural sites in Anapoima municipality, Colombia, October-December 2011. Both mosquito-positive and negative containers are included. Differences between urban and rural sites were tested using Chi-square test

Variable	Value	Urban (%)	Rural (%)	Chi-square	*P*
		*n* = 36	*n* = 35		
Container type	Wash basin (*alberca*)	52.8	60.0	0.38	0.540
	Ground tank	47.2	40.0		
Location of container	Outdoors	52.8	73.5	3.22	0.073
	Indoors	47.2	26.5		
Material of container	Plastic	33.3	20.6	6.31	0.043
	Cement	55.6	79.4		
	Metal	11.1	0.0		
Source of water	Village pipe	5.6	62.9	40.33	< 0.0001
	Municipal pipe	61.1	5.7		
	Rain only	11.1	11.4		
	Rain + village pipe	8.3	17.1		
	Rain + municipal pipe	13.9	0.0		
	Village pipe + river + spring	0.0	2.9		
Use of water	Washing + cleaning	58.3	60.0	0.32	0.956
	Multiple uses, including drinking	11.1	11.4		
	Multipe uses, excluding drinking	25.0	25.7		
	Drinking only	5.6	2.9		
Lid status	Effective lid	22.2	8.8	2.42	0.299
	None	58.3	70.6		
	Ineffective lid	19.4	20.6		
Frequency of container washing	Weekly	27.8	48.6	3.94	0.140
	Monthly	52.8	31.4		
	Never	19.4	20.0		
Location in shade	Yes	50.0	38.2	1.08	0.584
	No	16.7	23.5		
	Partial	33.3	38.2		
Location under roof	Yes	55.6	55.9	2.58	0.275
	No	13.9	26.5		
	Partial	30.6	176		
Algae in container	Present	19.4	22.9	0.12	0.725
Organic material in container	Present	66.7	42.9	4.06	0.044
Macroinvertebrates in container	Present	19.4	5.7	3.02	0.082

**Table 5:** Container and water characteristics (continuous variables) in urban and rural sites in Anapoima municipality, Colombia, October-December 2011. Both mosquito-positive and negative containers are included

Variable		Urban	Rural	*df*	*t-*value	*P*
1	Water temperature (°C)	25.5 (25.1–26.0)	22.7 (22.3–23.0)	69	-10.36	< 0.00001
2	pH	7.42 (7.18–7.66)	7.72 (7.50–7.94)	69	1.87	0.033
3	Total dissolved solids, TDS (mg/l)	100.2 (85.6–114.9)	58.7 (37.9–79.4)	69	-5.14	< 0.00001
4	Electrical conductivity, EC (μS/cm)	152.6 (128.2–176.9)	91.3 (58.2–124.4)	69	-4.31	< 0.00001
5	Ammonia, NH_4_ (mg/l)	0.30 (0.26–0.33)	0.24 (0.20–0.28)	69	-1.71	0.046
6	Phosphate, PH_4_ (mg/l)	0.13 (0.10–0.17)	0.16 (0.11–0.22)	69	0.96	0.339^a^
7	Nitrate, NO_3_ (mg/l)	0.42 (0.37–0.47)	0.22 (0.16–0.29)	69	-5.49	< 0.00001
8	Dissolved oxygen, DO (mg/l)	6.25 (5.97–6.53)	5.85 (5.42–6.29	69	-1.56	0.124^a^
9	Total suspended solids, TSS (mg/l)	8.03 (4.80–11.25)	45.86 (11.66–82.05)	69	4.40	< 0.00001
10	Number of bacteria (log_10_ bacteria /ml)	5.00 (4.81–5.23)	5.06 (4.53–5.58)	33	0.25	0.804^a^
11	Altitude (masl)	697.3 (689.5–705.1)	1037.9 (1022.0–1053.8)	68	39.80	< 0.00001

Differences in mean values (95% CI) between urban and rural sites were tested using Student’s t-test (*df* = degrees of freedom). The results of the t-tests of TDS, EC, NH_4_, PH_4_, NO_3_, and TSS are based on log10-transformed data, although the urban and rural values presented are the actual means for asier comparisons

^a^Two-tailed test

## Discussion

*Aedes aegypti* bred in containers in both urban and rural settings, but the productivity of pupae was higher in urban containers, where the odds of pupal infestation was five times higher (*P* = 0.03). In this study, mosquitoes were only collected from ground tanks and washbasins, because these are most productive containers [32]. Other containers, such as rubbish and tires, etc. may produce low numbers of mosquitoes, but usually only in the rainy season [21, 33]. Although these collections were done in the rainy season (October–December), the main mosquito producing permanent breeding habitats would be the most important to determine differences between urban and rural mosquito production. A limitation of the study design, however, was that containers were selected on the basis of being positive or negative, with equal numbers of each being included. Hence, we could not compare the proportion of *Aedes-*positive houses or containers, between urban and rural areas, in terms of indices such as the Breteau. The numbers of positive or negative containers which were inspected, but not included to preserve balance, could have been used as weights in a more informative analysis, but these numbers were unfortunately not recorded.

Considering the higher density of people, houses, and water storage containers in urban compared to rural settings, it is likely that *A. aegypti* productivity and dengue risk are higher in the former setting. Nevertheless, the current data show that *A. aegypti* rural breeding is substantial and should not be ignored. Dengue transmission and outbreaks, as well as DENV infected mosquitoes are not that uncommon in rural areas [9, 16, 59]. Adult *A. aegypti* collected in 2012–2013 in the rural study area showed high DENV infection rates, with a pool positivity rate of 62% and estimated individual mosquito infection rate of about 4% [16]. These facts indicate that rural areas are at substantial risk of dengue, and therefore merit regular entomological surveillance to detect locations for effective vector control interventions.

One of the most important factors associated with *A. aegypti* infestation was the frequency of container washing, both in urban and rural settings. Containers reported to be washed every month were more than four times more likely to be infested with *A. aegypti* than those reportedly washed every week. Never-washed containers were also four times more likely to be infested than those washed every week. This suggests that cleaning washbasins and low tanks on a regular basis is an effective mosquito control activity. The importance of cleaning containers for dengue vector control has been shown in many studies [60–63] and is recommended by WHO [64]. By promoting the use of detergents and/or chlorine and brushing the inside walls of containers, community dengue vector control using these methods has been shown to be effective [65]. In Colombia, the *alberca*, a square concrete laundry basin, is ubiquitous and very often mosquito-infested [15, 66]. Most have two sections, one for storing water and the other for washing clothes with horizontal grooves in the concrete. This design makes it difficult to inhibit mosquito breeding. New washbasin designs and technologies could be an effective way to reduce mosquito breeding in settings as those studied here [66].

Other factors of potential importance for *A. aegypti* infestation were the concentration of TDS and DO in water. TDS was positively associated with *A. aegypti* infestation in the univariate model (Table 2), but was not included in the multivariate model (Table 3). TDS is the sum of inorganic salts and small amounts of organic matter dissolved in water and is thus a measure of the combined content of all inorganic and organic substances contained in a liquid in molecular, ionized or micro-granular suspended form. Such water may contain particles which constitute food and nutrients for developing larvae [20, 44]. The mean TDS values in urban and rural containers (urban: 100 mg/l; rural: 59 mg/l, Table 5) were low in comparison to other studies. For example, the TDS in natural stream water from mountain sites and valley sites were ~200 mg/l and 400–600 mg/l, respectively [67, 68]. Likewise, TDS in *Aedes* breeding habitats in West Bengal, India were all higher than 200 ppm [69], i.e. close to stream water values. This could be explained by more variable types of breeding habitats included in the India study, such as earthen containers, coconut shells, tires, tree holes, and plastic containers. Also in these containers, there was a significant positive correlation between TDS and larval density [69]. It may, therefore, be that TDS is a contributing factor to breeding success, but, when other factors are taken into consideration (multivariate results), this factor may become less important.

DO was negatively associated with *A. aegypti* infestation in both models (Tables 2 and 3), but there were no differences in DO levels between urban and rural sites (Table 5). Ma et al. [70] also found a significantly negative association between DO and overall larval abundance in Chinese urban river systems, although these larvae mainly consisted of *Culex* species. On the other hand, *A. aegypti* has been observed to oviposit and develop normally in raw sewage with low levels of dissolved oxygen, suggesting a wide tolerance spectrum [36]. However, this is probably more the exception than the norm. It is also worth noting that DO fluctuates on a daily and seasonal basis, sometimes as much as from 1 to 20 mg/l, based on variations in natural processes such as diffusion, aeration, photosynthesis, respiration, decomposition, temperature, and air pressure [71]. The magnitude of such variations in water storage containers is unclear. Nevertheless, it is reasonable to assume that lower oxygen content reduces the suitability for mosquito immature development, as has been shown for several mosquito species [72] and other aquatic invertebrates [73].

The association between non-*Aedes* larvae (mostly *Culex*) and urban/rural settings was opposite to that of *Aedes* mosquitoes, with urban containers less likely to be infested. The reasons for this are unclear. *Culex* species are generally considered to be more associated with contaminated waters, relative to *A. aegypti* [74]. In this study, the water in the urban containers seemed to be more contaminated than the rural containers, with higher values of EC and TDS, and higher content of ammonium and nitrate, which, at least, would indicate a higher degree of particulate matter and organic material. However, the overall levels of TDS, EC, and nitrate were low compared to other studies [68, 75, 76] and differences in these parameters do not explain all of the variation in *Culex* infestation. *Culex* spp. are ubiquitous mosquito species adapted to many types of environments, such as rural, urban, clean and contaminated water (e.g. [77]) and might have adapted well in this rural setting. Our data do not clearly explain why *Culex* spp. were more abundant in these rural settings.

Water temperature, another important factor for mosquito metabolism and development, was significantly higher in urban than in rural containers. Water temperature is affected by the surrounding ambient temperature, which is higher at lower altitudes and is also affected by urban settings, due to human activities and modification of land surfaces, a phenomenon called urban heat island [78]. The urban households in this study were generally located at a lower altitude than the rural households, potentially affecting ambient, as well as water temperatures. Mosquito development is faster in warm water than in cold water and maximum survival rates being at 20–30 °C [79]. The temperatures in this study were within these limits. Higher ambient and water temperatures may affect mosquito population dynamics, potentially reducing development times and increasing mosquito production [80–83]. Water temperature could be expected to affect both *Aedes* and *Culex* production similarly, but this was apparently not the case. Nevertheless, these results show that vector control is important in rural as well as urban areas, not only for dengue, but also for other vector-borne diseases, such as those transmitted by *Culex* and other genera. For example, Olano et al. [10, 15] found several potentially important mosquito vector genera, in addition to *Aedes*, in rural schools in the same study area.

As the characteristics of the water are important to immature development, they also affect the oviposition behavior of female mosquitoes. Ovipositing *A. aegypti* are affected by the bacterial composition of water [84], presence of conspecifics [41], and other potential cues from the water and the container in order to maximize survival of offspring. Such factors may explain the skip oviposition behavior of this mosquito species [40]. It is possible that these small, though significant physicochemical differences could have favored *A. aegypti* production in urban over rural containers. On the other hand, another study from India found opposite results to ours, with a negative correlation between TDS and larval density and a positive correlation between DO and larval density [75]. This suggests that it is difficult to find universal agreement on specific physicochemical characteristics that are most important for *A. aegypti* breeding success and production. Risk of dengue has many other determinants including adult vector density and production, serotype circulation, human population density, movement, behaviors and immunity. Therefore, although control of vector breeding sites will remain an important part in arbovirus control, it alone may not reduce the disease burden of arboviruses, such as dengue, Zika, and chikungunya.

## Conclusions

Mosquito vectors, such as *A. aegypti* and *Culex* species breed in containers in both urban and rural settings, but urban containers were more likely to be infested with *A. aegypti* immatures than rural containers. In contrast, rural containers were more likely to be infested with *Culex* immatures. It should be noted though, that only ground tanks and washbasins were sampled in this study, as they currently are considered the most productive containers in both urban and rural areas. As mosquito ecological relationships are dynamic, such assumptions may change due to environmental and climate change. Containers that were washed more frequently (weekly or monthly) were less likely to be infested with *A. aegypti* than containers that were never washed. *Aedes aegypti* immature infestation was positively associated with total dissolved solids, but negatively associated with dissolved oxygen. Recommendations based on these results are that vector control should not only be carried out in urban areas, which is often the case, but also be implemented in rural areas. A suitable and effective vector control option in these settings is frequent container washing. Development of alternative designs of the highly mosquito productive washbasins in Colombia should continue to be explored.
